# Misdiagnosis of Hepatosplenic Schistosomiasis as Hepatic Tuberculosis: A Case Report

**DOI:** 10.7759/cureus.35169

**Published:** 2023-02-19

**Authors:** Joselv E Albano, Ralph Benedict B Ma-alat

**Affiliations:** 1 Department of Surgery, Southern Philippines Medical Center, Davao City, PHL; 2 Institute of Radiology, St. Luke's Medical Center, Quezon City, PHL

**Keywords:** pipestem fibrosis, schistosomiasis, case report, hepatic tuberculosis, misdiagnosis, hepatosplenic schistosomiasis

## Abstract

We report a case of a 38-year-old woman who was initially misdiagnosed with hepatic tuberculosis and was managed as such before being correctly diagnosed with hepatosplenic schistosomiasis on liver biopsy. The patient had a five-year history of jaundice which over time was accompanied by polyarthritis and then abdominal pain. A diagnosis of hepatic tuberculosis was made clinically and supported by radiographic evidence. She underwent an open cholecystectomy for gallbladder hydrops with the liver biopsy taken revealing chronic hepatic schistosomiasis and was eventually started on praziquantel with good recovery. This case demonstrates a diagnostic issue with the radiographic presentation of the patient and the important role of tissue biopsy in providing definitive care.

## Introduction

Schistosomiasis is a parasitic infection caused by the genus *Schistosoma*. This disease is estimated to affect at least 230 million people worldwide [1] with as much as 5.8% of chronic infections presenting with the complication of hepatosplenic schistosomiasis [2]. Chronic infection leads to granuloma formation around embolized eggs in the liver culminating years later as periportal fibrosis [3]. Complications arising from this include presinusoidal portal hypertension and its sequelae of splenomegaly, portosystemic collateral and variceal formation, and ultimately the most dreaded complication of upper gastrointestinal bleeding [3,4]. 

Here we present a case of a woman with an ex-juvantibus misdiagnosis of hepatic tuberculosis managed with the standard anti-tuberculosis regimen. Her clinical and radiographic presentation, with computed tomography (CT) scans revealing multiple parenchymal hepatic calcifications, was congruous with hepatic tuberculosis. She had multiple admissions and outpatient visits with the management centered on anti-tuberculosis therapy for the past five years. It was only until a biopsy of a liver lesion was done that a diagnosis of hepatosplenic schistosomiasis was made and an appropriate therapy was initiated.

## Case presentation

Patient information

The patient is a 38-year-old housewife from Davao de Oro, Philippines presenting with a five-year history of waxing and waning jaundice. She did not have any notable history of prior diseases, did not drink alcohol or smoke, and was with an unremarkable family history. At the initial consultation five years prior, the patient presented with weight loss, insomnia, and cough with no fever or other symptoms. Though sputum smear microscopy tested negative for tuberculosis, chest x-ray, abdominal ultrasonography, and abdominal CT scan done at that time led to the diagnosis of hepatic tuberculosis with concurrent pulmonary infection. She was managed with a standard anti-tuberculosis regimen with rifampicin, ethambutol, isoniazid, and pyrazinamide in addition to ursodeoxycholic acid for six months. She recovered well regaining her lost weight with a notable decrease in jaundice. 

In the interim, she was well until one year prior when jaundice recurred with associated severe polyarthritis affecting all four girdles and the extremities in a symmetric fashion. There was no associated fever, cough, weight loss, or other symptoms. Repeat ultrasound and CT imaging revealed the same hepatic lesions but with an incidental burst fracture in the lumbar area and was managed as tuberculosis of the bone and was started on a 12-month anti-tuberculosis regimen with full compliance and resolution of symptoms. However, a few months after the completion of this treatment regimen, her jaundice started to worsen, now with vague epigastric pain, vomiting, and occasional febrile episodes. She then consulted at our center where she was then admitted as a case of hepatic tuberculosis. A repeat CT scan showed the same hepatic lesions now with splenomegaly, and gallbladder hydrops with cholecystitis. Throughout this history, both CT scan and ultrasonography did not show signs diagnostic of schistosomiasis.

Clinical findings

On admission, the patient appeared frail with generalized jaundice. She was febrile as high as 38.2°C, tachycardic with a heart rate of 110 beats per minute, with other hemodynamic parameters normal. The abdomen was soft, and slightly distended, with only mild tenderness on direct palpation of the right upper quadrant, and there was no rebound tenderness. The abdominal examination was remarkable however for a palpable ill-defined right upper quadrant mass. Her breath sounds were clear on all regions of the chest. No lymphadenopathies were palpated. There was no recollection of possible infectious contacts. The rest of the physical examination was normal. The primary consideration at this time was acute cholangitis.

Diagnostic assessment

After initial management at the emergency department, the patient was transferred and received by the hepatopancreatic biliary service. Testing for hepatitis A, B, and C revealed negative results. Laboratory tests revealed elevated leukocytes, bilirubin, alkaline phosphatase, hepatic function markers (aspartate transaminase [AST], alanine transaminase [ALT]), and C-reactive protein consistent with an acute cholangitis profile (Table 1). A cartridge-based nucleic acid amplification test (GeneXpert) done on admission was negative for tuberculosis. HIV testing was also negative.

**Table 1:** Laboratory test results

Variables	Result	Reference Range
Alkaline phosphatase (U/L)	191.44	30.00-120.00
Aspartate transaminase (AST) (U/L)	64.7	<35.00
Alanine transaminase (ALT) (U/L)	52.8	<35.00
Total bilirubin (µmol/L)	300.62	5.00-21.00
Direct bilirubin (µmol/L)	148.79	<3.40
Indirect bilirubin	151.8	3.40-11.90
Albumin (g/L)	31.23	35.00-52.00
Creatinine (µmol/L)	56.4	39.0-91.0
C-reactive protein (mg/dL)	14.030	<1.000
Sodium (mmol/L)	136.0	136.0-146.0
Potassium (mmol/L)	2.9	3.50-5.10
Hemoglobin (g/dL)	12.7	12.1-15.1
Hematocrit (%)	35	36.0-45.0
Leukocytes (x10^3^/µL)	20.99	4.0-11.0
Neutrophil (%)	92	44-68
Lymphocytes (%)	5	20-40
Platelet (x10^3^/µL)	93	140-400
Prothrombin time (seconds)	23.9	11.5-14.3
Partial thromboplastin time (seconds)	32.5	27.7-32.9
International normalized ratio (INR)	1.82	1.00

**Figure 1:**
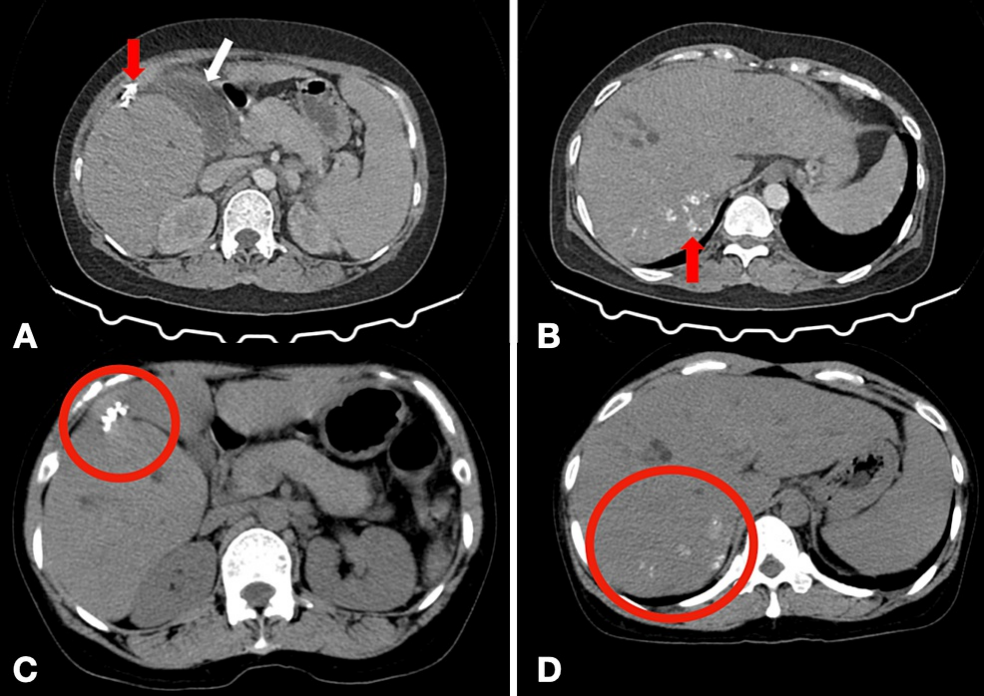
Parenchymal calcifications Coarse calcifications are identified in hepatic segment V (Figure 1A, red arrow) and segment VII (Figure 1B, red arrow). The gallbladder in Figure 1A (white arrow) is distended with minimal surrounding fluid and stranding densities compatible with gallbladder hydrops and cholecystitis. Similar calcifications (red circles) are noted 4 years prior on the same lobes of the liver (Figure 1C, segment V) and (Figure 1D, segment VII) demonstrating a significant increase in the size of the calcifications.

We decided to proceed with a contrast-enhanced CT scan due to her clinical and imaging history. The liver was noted to be enlarged but with normal parenchymal density except for irregular calcific densities in segments V and VII (Figure 1A, 1B). Comparison with the patient’s abdominal CT scans and abdominal ultrasound done four and five years prior (Figure 1C, 1D) revealed the same parenchymal calcifications but with a significant increase in those located at segment VII consistent with hepatic tuberculosis. There were no other discrete enhancing mass lesions or other abnormal parenchymal densities identified. The intrahepatic bile ducts remained dilated as before. The gallbladder was now dilated measuring 8.0 cm x 5.0 cm with an associated minimal pericholecystic fluid collection compatible with hydrops and cholecystitis. The common bile duct was enlarged (9 mm) secondary to extrinsic compression by enlarged calcified hepatic perihilar lymph nodes. Splenomegaly was also observed. The portal vein was not dilated and there were no collateral vessels to suggest portal hypertension.

**Figure 2:**
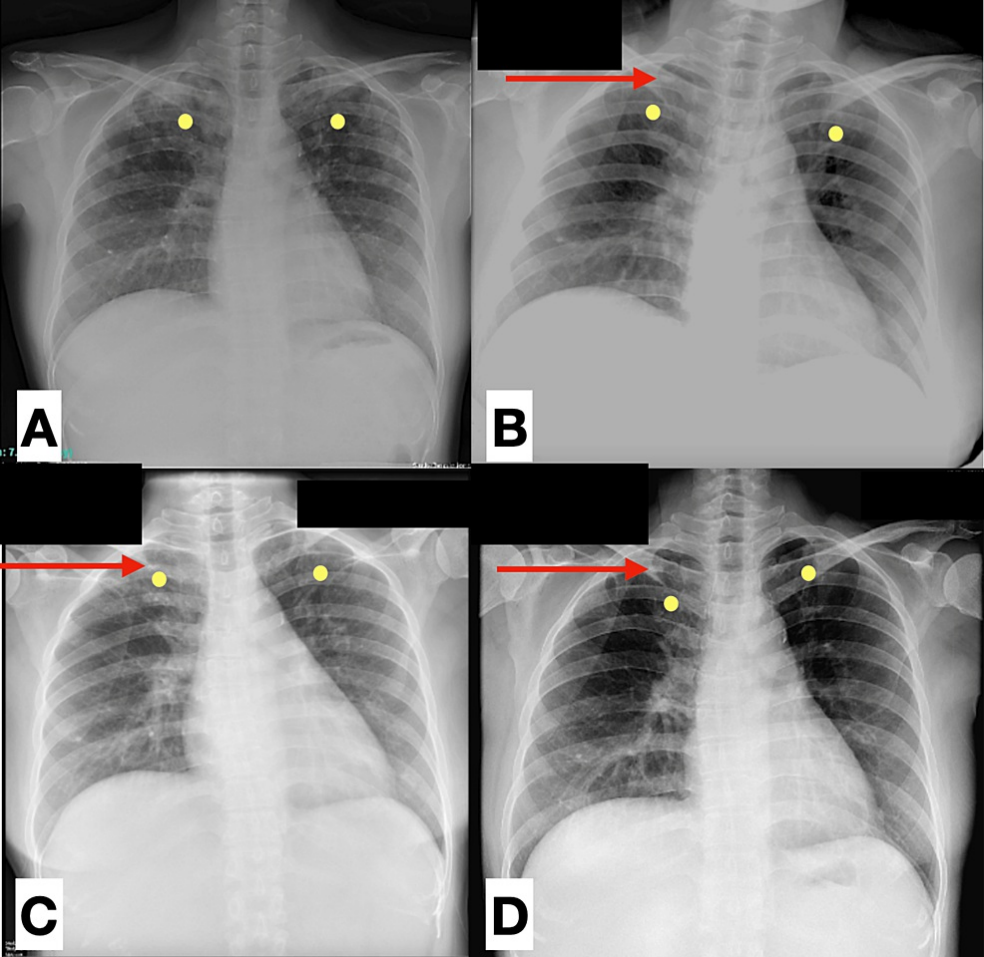
Chest x-rays Chest x-rays were obtained at five years prior (A) , three years prior (B), one year prior (C), and admission (D). The hazy densities (yellow circles) in both upper lungs are noted to be regressing on subsequent chest x-rays with only few remaining densities at the time of admission (D). The small ovoid density on the right upper lung (red arrow) was first seen three years prior (B) and appears stable in subsequent chest x-rays done in (C) and (D), likely relating to a pulmonary tuberculosis with granuloma formation.

Chest x-ray done on admission demonstrated regressing hazy densities on both upper lungs. A small ovoid density on the right upper lung was also identified and appeared stable when compared with the patient’s previous chest x-rays (Figure 2). These findings were consistent with bilateral pulmonary tuberculosis with granuloma formation on the right.

No preoperative liver biopsy was done for reasons of tuberculosis endemicity, financial restrictions, and patient preference. A diagnosis of obstructive jaundice secondary to extrinsic common bile duct compression secondary to calcified hilar lymphadenopathy probably secondary to hepatic tuberculosis in acute cholangitis with gallbladder hydrops was made.

Therapeutic intervention

Upon admission, the patient was placed on nothing by mouth and started on 40 mg omeprazole once a day, 10 mg hyoscine n-butyl bromide every 8 h, and 50 mg tramadol intravenously (IV) every 8 h to control the abdominal pain. She was hydrated with plain lactated ringers 1 L 100 cc/h. The hypokalemia was subsequently corrected. Resolution of abdominal pain was noted 8 h after initial management at the emergency department. 

**Figure 3:**
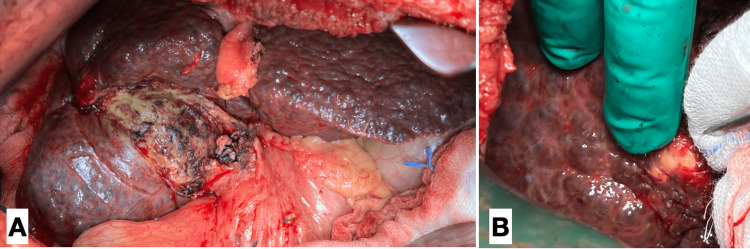
Gross liver anatomy and calcified hepatic nodule (A) Grossly nodular liver (post-cholecystectomy) contour consistent with a case of hepatosplenic schistosomiasis reported by Bolgiolo in 1957 [5]. (B) A yellow-white-colored calcified hepatic nodule identified on segment VII.

**Figure 4:**
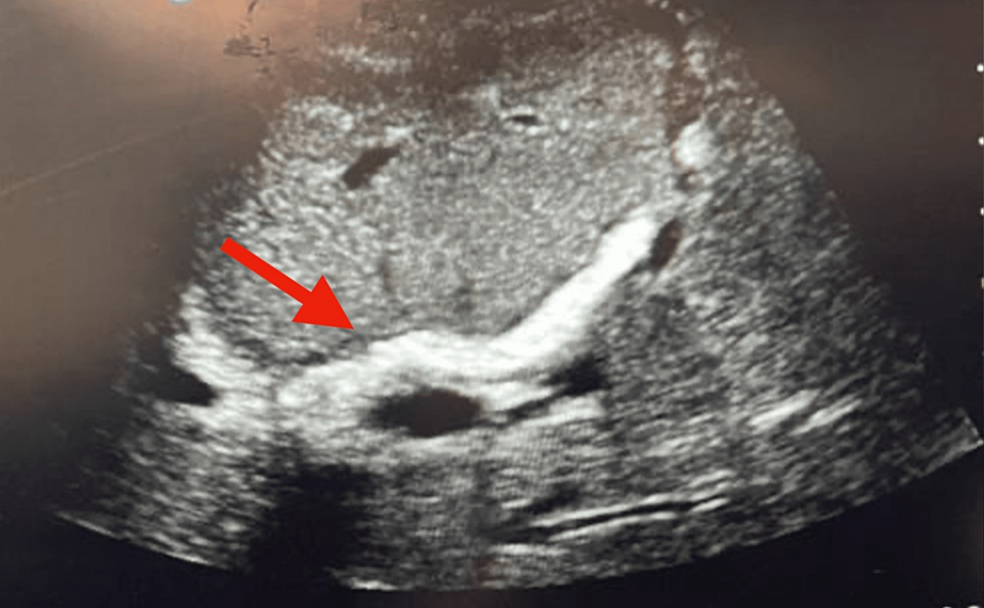
Symmers pipestem fibrosis Intraoperative ultrasonography revealed prominent portal spaces with surrounding hyperechoic areas (red arrow) reflective of Symmers pipestem fibrosis, a characteristic finding in advanced stages of hepatic schistosomiasis.

The patient was maintained on ceftriaxone 2 g IV once daily and metronidazole 500 mg IV every 8 h. This was continued throughout the hospital stay of the patient. Due to the presence of gallbladder hydrops, the patient consented to proceed with operative intervention. She then underwent an open cholecystectomy via a Kocher’s incision along with an intraoperative sonogram. Intraoperatively, the liver was noted to have a diffuse nodular appearance (Figure 3A) with a yellow-white-colored calcified nodule identified on segment VII (Figure 3B) consistent with a case of hepatosplenic schistosomiasis reported by Bolgiolo in 1957 [5]. Biopsy samples of the calcified hepatic nodule, the peritoneal wall, and the omentum were taken. Intraoperative ultrasound revealed the characteristic thickened portal tracts (Figure 4) resembling Symmers pipe stem fibrosis in advanced hepatosplenic schistosomiasis [6]. She was then started on ursodeoxycholic acid 300 mg orally every 8 h to counteract further damage to what appeared to be a chronically damaged liver.

Follow-up and outcome

**Figure 5:**
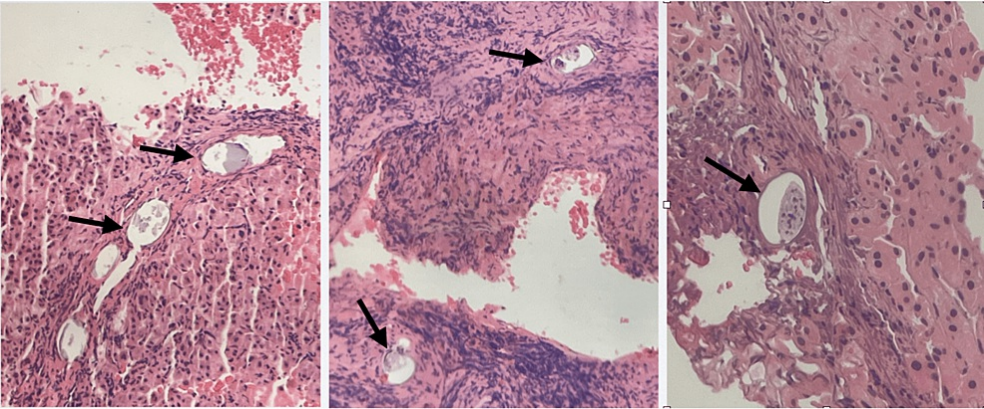
Liver tissue biopsy of the hepatic nodules All sampled tissue from hepatic nodules revealed multiple *Schistosoma* eggs (arrows) establishing the diagnosis of hepatosplenic schistosomiasis.

The postoperative period was unremarkable with subsequent discharge after one week. The patient had improving jaundice during the course of the hospital stay with declining bilirubin levels. The patient was seen again after one month with the results of the biopsy (Figure 5) confirming schistosomiasis. The patient during this period had two episodes of melena with minimally improved jaundice. Upper gastrointestinal endoscopy was done revealing esophagitis with few mucosal breaks <5 mm and a duodenal ulcer measuring 1.5 cm at the D1 segment classified Forrest III, and the urease test was negative. Repeat testing was remarkable for a new hepatitis B infection. The patient refused further intervention. She was discharged with instructions for yearly praziquantel 500 mg every 8 h for three doses, omeprazole 40 mg twice a day for 14 days, sucralfate 1 g every 6 hours for five days, and tenofovir 300 mg daily. The goal of treatment is to reduce worm load, but at this advanced stage of the disease, only a minimal effect is expected in terms of reversing the hepatic insult. She had no further medical complaints since this visit.

## Discussion

Hepatic tuberculosis was seen as the most likely diagnosis given the history, physical examination, and radiologic findings of the patient. Calcified liver lesions are usually due to inflammatory processes, most commonly due to granulomatous disease [7]. In endemic regions, tuberculosis is the most common cause of hepatic granuloma formation [8-10]. The combination of chest x-ray findings compatible with tuberculosis made it logical to diagnose the case as hepatic tuberculosis given that the Philippines is a known endemic region [11]. The claimed response of the patient to anti-tuberculosis medication also cemented this idea. It should also be remembered that the patient concurrently was given ursodeoxycholic acid, which could explain the clinical improvement of her jaundice. Radiographically, regression was documented with pulmonary tuberculosis but not with the supposed hepatic tuberculosis. At this point in the management, further investigation should have already been done when the hepatic lesions continued to progress despite intensive anti-tuberculosis therapy. With this epidemiological background as well as the financial constraints faced by patients, it has not become routine to biopsy hepatic lesions whose imaging appearance is consistent with hepatic tuberculosis. Looking back, the patient’s polyarthritis could also be attributed to schistosomal infection as osteoclast-mediated bone loss is a known immunological consequence [12].

The key factor for this case, however, was the locality, Davao de Oro, Philippines, from which the patient originally came from before moving to the city. This region in the Philippines is known to be endemic to schistosomiasis [13]. At the time of the first consult, the patient was already living in the city with no significant history of travel or exposure hence implying the chronicity of the infection. 

Parenchymal hepatic tuberculosis can present in macronodular and micronodular forms which can calcify and present as hepatic calcifications on imaging [14,15]. Hepatosplenic schistosomiasis also presents with calcifications on the liver owing to the embolized eggs and the granulomatous inflammation that occur around it. This was not enough, however, to stir the case toward schistosomiasis as it lacked the typical CT imaging findings of the disease such as concentric layers of periportal enhancement usually enhancing strongly on contrast, septal enhancement in broad fibrous septa, as well as septal calcifications that resemble a turtle shell appearance [16]. It is then understandable that there is some difficulty in differentiating these two entities for this case. The confusion arises from the fact that both may present as hepatic calcifications in the background of a plethora of both pulmonary and extrapulmonary tuberculosis cases in the Philippines [17]. 

Infection by *Schistosoma japonica, S. mansoni, *and* S. mekongi* is known to commonly cause this infection due to granuloma formation around embolized eggs that are lodged in the hepatic portal venules [3]. This eventually led to an inflammatory response that culminated years later as periportal fibrosis pathognomonically referred to as Symmers “pipestem” fibrosis and was evident on the patient’s intraoperative ultrasonography [6]. Complications arising from this fibrotic process are due to the presinusoidal blockage to portal blood flow that eventually leads to presinusoidal portal hypertension and its corresponding sequelae of splenomegaly, formation of portosystemic collaterals and varices, ultimately to the most dreaded complication of upper gastrointestinal bleeding [3,4]. 

The accuracy of using a CT scan to detect periportal fibrosis in hepatic schistosomiasis has not been documented while ultrasound, on the other hand, has been cited to have a 97% sensitivity and 95% sensitivity [18]. It is interesting to note, however, that periportal fibrosis was not seen in any of the ultrasonographic studies done prior to the admission. Indeed, a sonographic correlation in retrospect, which was not done on this present admission, could have helped document the now evident periportal fibrosis characteristic of the disease seen intraoperatively [19]. The admitting team, however, did not proceed with this imaging choice since previous ultrasounds did not yield significant clinical findings, except for hepatic calcification. This explains why the primary choice at the time of the admission was a CT scan. This highlights the importance of routine point-of-care ultrasound proficiency in aid of diagnosis and management for the surgical professional. Finally, it should be accepted that the history and physical examination even with imaging and laboratory tests can still have limitations. This case report demonstrates the invaluable role of a biopsy in the management of patients. An early diagnosis for this patient could have significantly improved her outcomes as well as decrease the economic, psychological, and physical burden of the disease.

## Conclusions

This case report demonstrates the invaluable role of tissue biopsy in the definitive management of patients. These two disease entities, tuberculosis and schistosomiasis, were both endemic in the patient's locality. As both these infections potentially present with hepatic lesions, tissue biopsy would have been the best option rather than purely relying on imaging diagnosis. As outcomes could have significantly improved with early diagnosis, management should be based on more reliable diagnostic data given this dilemma rather than an ex-juvantibus diagnosis. 
